# Applications of Whole Brain Tractography and Implications for Clinical Practice

**DOI:** 10.7759/cureus.1753

**Published:** 2017-10-05

**Authors:** Brian W Wu, Stephanie Barr

**Affiliations:** 1 Keck School of Medicine, University of Southern California; 2 Division of Children with Special Needs, Heart of the Ozarks Healthcare Center

**Keywords:** whole brain tractography

## Abstract

The complicated nature of neurological diseases–and the importance of accurate diagnosis and treatment for patient quality of life–have made the need for more advanced imaging techniques more urgent than ever. Automated whole brain tractography promises to increase the knowledge that neurologists have of a variety of disease processes, including schizophrenia, age-related changes to white matter, brain tumors, and epilepsy.

## Introduction and background

Researchers note that “the clinical importance, structural fragility, and organizational complexity” of the brain require a high degree of skill and advanced technology in order to further understanding of its structure, function, and development [1]. The importance of imaging techniques in neurology cannot be overstated. Neuroimaging has contributed to an increased understanding of the classifications, localizations, pathology, and disease progress of a variety of neurological disorders. This increased knowledge can facilitate the formation of clinically testable hypotheses and, ultimately, the development of new therapeutic modalities [2].

## Review

The importance of imaging and automation

There are several medical imaging techniques that help to reconstruct and, thus, gain a deeper understanding of the structure of the white matter of the brain. Tractography is foremost among these techniques, but, in the past, this was done on a manual basis. This technique was both time-consuming and required a high level of staff expertise. Automated versions of this technique can help eliminate both of these shortcomings [3].

The specificity of whole brain tractography seeks to go beyond the limitations of other imaging methods currently available in neurosurgery. One such method is diffusion tensor imaging (DTI), which is only able to study one fiber at a time and cannot model fiber crossings. Functional MRI (fMRI) and diffusion MRI (dMRI) techniques can be difficult to translate and are not suited for widespread clinical use. In addition, the dMRI technique requires a high degree of expertise when it comes to the matter of selection of tracts and interpretation of results, making it difficult to standardize the technique [4].

Specifically, O’Donnell notes that the goals of automated identification/mapping include:

1. Decreased clinical time needed for human interaction

2. Increased standardization of pre-surgical planning

3. Reduced operator-dependent effects such as choice of seeding or selection region

Knowledge of the process of tractography techniques is still emerging. Chen and fellow researchers note that what is still lacking is a comprehensive list of tract atlas and an accurate registration method. Researchers were able to help fill this knowledge gap through reconstructing 76 major white matter tracts on a diffusion spectrum imaging (DSI) template and then using a two-step registration method, which combined information on white matter from diffusion-weighted images, using automatic methods to establish a transformation between the DSI template and the DSI data set. A validation study of this method on eight well-documented tracts found that the automatic tractography technique had “high geometric agreement with manual tracts in both deep and superficial parts but significantly smaller measurement variability” [3].

Applications for whole brain tractography

One particularly intriguing aspect of whole brain tractography for researchers is its wide range of possible applications for a variety of disease processes, including schizophrenia, epilepsy, brain tumors, and age-related white matter changes in the brain.

Schizophrenia: Researchers have noted that an increased understanding of schizophrenia can help facilitate a better understanding of the genetic underpinning of the disease and that microstructural integrity of white matter tracts could be seen as a trait marker of schizophrenia. To this end, tractography-based automated analysis (TBAA) is the current method of choice. The study used TBAA to identify a trait marker from among 74 fiber tracts across the whole brain in order to better understand its genetic components [2].

In the face of increasing evidence that abnormal connections in the brain can play a part in this mental illness, researchers used TBAA methods on 31 patients with chronic schizophrenia, 31 unaffected siblings, and 31 healthy controls. A total of 74 tracts were studied and 10 were found to vary significantly from one group to another. Analysis showed that, in particular, generalized fractional anisotropy of the right arcuate fasciculus was significantly decreased in both schizophrenia patients and their unaffected siblings. The study also uncovered the fact that there was a difference between these tracts in those whose schizophrenia treatment was successful and those whose treatment was not. Researchers noted that “altered tracts renewed by TBAA might become potential biomarkers or trait markers for schizophrenia” [5].

Other researchers have used automated whole brain tractography to gain a deeper understanding of the altered connectivity in the brain, which can be linked to schizophrenia. This study looked at 31 chronic schizophrenics, 25 first-episode schizophrenics, and 31 healthy controls in an analysis of 74 different fiber tracts. They found that there was a significant variation in seven different fiber tracts between the three groups of patients. One finding that emerged was that the connection of the callosal fibers to the bilateral dorsolateral prefrontal cortices as well as the bilateral hippocampi and temporal poles showed significant deterioration between the chronic and first-time schizophrenics, indicating the possibility that these connections degenerate as the disease progresses [6].

Brain Tumors: Another important potential application for TBAA methods is for the surgical treatment of brain tumors. Preservation of sensory, motor, visual, language, and other functions of the brain is an important consideration in neurosurgical planning, and an understanding of white matter and fiber tract connections is useful for this, so that surgery might spare as much cortical function as possible. In the O’Dell study, researchers note that neurosurgeons can face an array of challenges when it comes to tumor excision, including displacement, edema surrounding the tumor, and the mass effects caused by the tumors themselves [4].

Scientists used a new tractography method to help overcome these challenges by creating a fiber cluster atlas and identifying key fiber tracts in healthy control subjects than by using automatic methods to identify fiber tracts in brain tumor patients. Results were that 80% of the 800 fiber clusters were identified in all 18 patients and 94% of those clusters were found in at least 16 of them. Researchers noted that “Overall, our research indicates the potential of an automated method for identifying fiber tracts of interest for neurological planning, even in patients with mass lesions” [4].

Epilepsy: Increasingly, there is evidence to show that alterations in the white matter of the brain can affect important aspects of epilepsy, including its prognosis, chronicity, severity, and the extent to which the hippocampus has become atrophied. Researchers have applied automatic tractography methods in an attempt to quantify these white matter alterations and to gain a deeper understanding of seizure onset location and the resulting cognitive impairment from these seizures. The results of the application of these automated tractography methods revealed significant alternations in white tract matter between epilepsy subjects and controls, particularly in the ipsilateral inferior-longitudinal, uncinate fasciculus, superior longitudinal fasciculus, and cingulum [7].

Other researchers have also used automated tractography methods to study temporal lobe epilepsy (TLE). In this study, scientists looked at the differences in brain structure between right TLE and left TLE patients. They found significant differences in a number of the fiber tracts and posited the theory that right and left TLE might be distinct disease processes that cause their own unique changes to the structure of the brain [8].

Age-Related White Matter Changes to the Brain: Tractography methods have also been used in order to gain a greater understanding of just how the process of aging affects white matter tracts in the brain, specifically the presence of lesions and loss of tissue with corresponding ventricular enlargement. Scientists have used an automatic tractography method, known as probabilistic neighborhood tractography (PNT), on 90 non-demented patients over the age of 65. They established a negative relationship between age and the fractional anisotropy (FA) of the corpus callosum genu as well as a positive relationship between age and the mean diffusion of the bilateral cingulum cingulate gyri, the right corticospinal tract, and the unicate fasciculi [9].

Research is underway at the University of Aberdeen to use the automatic whole brain tractography method to better understand age-related neurodegenerative diseases, such as Alzheimer's disease, by increasing their knowledge of normal, age-related white matter changes that take place in the brain. To this end, TBAA methods will be applied to identify normal white matter microstructural functions and to identify regions where age is significantly associated with microstructural features in order to increase knowledge of age-specific normal adult brain templates [6].

## Conclusions

Neurological issues, including schizophrenia, epilepsy, brain tumors, and the changes that the brain undergoes in the normal process of aging, can prove challenging to treat and can negatively impact patients with these diagnoses. Better diagnostic tests, however, can lead to a deeper understanding of these conditions and even to more-effective and targeted treatments that can improve patient outcomes and quality of life.
